# Barriers and enablers to the use of seasonal climate forecasts amongst organisations in Europe

**DOI:** 10.1007/s10584-016-1671-8

**Published:** 2016-04-15

**Authors:** Marta Bruno Soares, Suraje Dessai

**Affiliations:** Sustainability Research Institute and the ESRC Centre for Climate Change Economics and Policy, University of Leeds, Leeds, UK

**Keywords:** Climate Information, Knowledge Broker, Public Sector Organisation, Advanced User, Climate Service

## Abstract

Seasonal climate forecasts (SCF) provide information about future climate variability that has the potential to benefit organisations and their decision-making. However, the production and availability of SCF does not guarantee its use in decision-making per se as a range of factors and conditions influence its use in different decision-making contexts. The aim of this paper is to identify the barriers and enablers to the use of SCF across organisations in Europe. To achieve that, we conducted 75 in-depth interviews with organisations working across eight sectors (including energy, transport, water and agriculture) and 16 countries. The majority of the organisations interviewed do not currently use SCF. This was due to the low reliability and skill of SCF in Europe but also with other non-technical aspects such as the lack of relevance and awareness of SCF in the organisations. Conversely, the main enabler to the use of SCF was the interactions with the providers of SCF. In addition, the level of organisational resources, capacity and expertise were also significant enablers to the use of SCF in organisations. This paper provides the first empirical assessment of the use of SCF in Europe. Such insights provide not only an overview of the existing barriers and enablers to the use of SCF in Europe and how these can be overcome and negotiated to enhance the usability of SCF, but can also help inform the broader and emerging context of climate services development in Europe.

**Electronic supplementary material:**

The online version of this article (doi:10.1007/s10584-016-1671-8) contains supplementary material, which is available to authorized users.

## Introduction

Adapting to, and managing the risks of, climate variability is crucial particularly in regions and economic sectors sensitive to climate conditions. Information about future climate variability can help to inform decision-making by providing a deeper understanding of the risks involved as well as supporting actions to reduce those risks (Troccoli et al. 2008). The availability of such information however, does not necessarily guarantee its use in decision-making processes (McNie 2007; Dilling and Lemos 2011; Feldman and Ingram 2009). In fact, the conventional linear model of science (also known as loading-dock model) where information is developed in the confinements of the scientific community with the expectation that users will find that information useful and usable has been challenged as ineffectual for decision-making (Feldman and Ingram 2009; Cash et al. 2006; Lemos 2015).

Sarewitz and Pielke (2007) argue the need to reconcile the supply and demand of science by bringing together scientists and decision-makers to frame and develop scientific information that is useful and usable for decision-making (McNie 2007). From a knowledge systems perspective Cash et al. (2003, 2005) defend the need for producing salient, credible and legitimate scientific information in order to make it ‘actionable climate knowledge’ (Meinke et al. 2006). Other contributions to this discussion include end-to-end systems (Agrawala et al. 2001) and co-production of science and policy (Lemos and Morehouse 2005). These underlying narratives permeate much of the discussion around the production of climate science and information and its use in policy and decision-making contexts.

Sitting between weather forecasts and climate change projections, seasonal climate forecasts (SCF) can appeal to, and benefit, a range of actors and economic sectors (e.g. agriculture, disaster risk management, health, water management, energy) (e.g. Patt et al. 2007; Archer et al. 2007; Barthelmie et al. 2008). These forecasts cover “the next month up to a year into the future” and the information is provided as monthly or seasonal means (Goddard et al. 2012; p. 622). As such, SCF provide a probabilistic estimate of how climatic parameters (e.g. temperature, rainfall) may develop in the coming months and thus can “(…) help to inform, focus and thus improve decision making” (Rickards et al. 2014; p.237). This in turn, can help to enhance operational activities, aid management processes, inform strategic planning, and increase profitability (Harrison et al. 2008; Rickards et al. 2014).

Recent scientific developments have led to improvements in SCF for Europe (Scaife et al. 2014; Doblas-Reyes et al. 2013). These include for example, the DEMETER and ENSEMBLES projects which aimed to develop multi-model ensembles for seasonal-to-annual forecasts (Palmer et al. 2004; Hewitt 2005; Weisheimer et al. 2009). In addition, the World Meteorological Organization has designated 12 Global Producing Centers^1^ which produce and provide operational long-range forecasts (from 30 days up to 2 years) three of which are based in Europe: the European Centre for Medium Range Forecasts, the Met Office in the UK, and Météo- France (Stockdale et al. 2010).

However, contrary to other regions (e.g. North America) where the influence of sources of seasonal predictability such as the El Niño-Southern Oscillation is stronger, in Europe the low forecast quality tends to makes it harder to understand the use that such forecasts have if any at all (cf. Doblas-Reyes et al. 2013). As a result, very little is known about how these forecasts are currently being used and the barriers and enablers pushing or limiting its use in Europe (cf. Bruno Soares and Dessai 2015). To improve existing knowledge, Bruno Soares and Dessai (2015) conducted a workshop with climate services providers and other scientific experts on this subject matter to elicit their knowledge and experience regarding their perceived use of SCF in Europe. They found that these experts perceived current use of SCF as quite limited and found in sectors such as energy, water, insurance and transport. Barriers to its use identified by these experts were mainly associated to the perceived low reliability of SCF but also with non-scientific aspects including lack of engagement and communication between the producers and users of SCF.

The aim of this paper is twofold: to identify the existing barriers to the use of SCF amongst European organisations and to identify the current drivers and enablers underpinning the use of SCF. In doing so the paper provides the first empirical assessment of the use of SCF in Europe.

Such insights can help understand not only possible ways of improving the development and production of SCF in Europe but also existing structural and organisational barriers that may be overcome to enhance the usability of these forecasts. Such knowledge is critical for the future development of a climate services market in Europe (European Commission 2015).

The next section presents conceptual frameworks from the scholarship underpinning the use of climate information. Section 3 describes the methods used to collect and analyse the data. Section 4 describes the barriers to the use of SCF in organisations not currently using these forecasts. Section 5 introduces the main enablers supporting the use of SCF as well as existing barriers that prevent a more involved and advanced use of SCF in those organisations. Section 6 discusses these barriers and enablers in relation to the wider conceptual frameworks presented in Section 2. Section 7 provides some conclusions.

## Usable climate information

Large contributions to the scholarship on the usability of climate information derive from critiques of the linear model of science. Simply put, this model (also known as Mode 1) assumes that basic research is developed by the scientific community and then applied by others to create products that (are expected to automatically) benefit society at large (Meyer 2011; Kirchhoff et al. 2013; Sarewitz and Pielke 2007). Allied to this idea is also the “common assumption that more [*climate*] information necessarily leads to better decision making or increased information use” (Meyer 2011, p. 51, emphasis added). These two key ideas have permeated much of the scientific research being developed which was primarily knowledge driven and based on what scientists perceived as useful or interesting science (Gibbons et al. 1994). However, albeit advancing scientific knowledge there has also been a wide spread recognition that the science produced was not supporting or informing decisions that could benefit from such knowledge (Kirchhoff et al. 2013; Meyer 2011).

Various frameworks have been developed to characterize new models of scientific knowledge production including Mode 2 and post-normal science. The former defines science as a reflexive, transdisciplinary, open and accountable; whilst in the latter scientific knowledge is considered as insufficient to deal with complex and uncertain societal problems (for more on these see e.g. Gibbons et al. 1994; Gibbons 2000; and Turnpenny et al. 2010, respectively).

Overall, and underpinning much of the discussion around the production of science and the usability of climate information, are the two central ideas that scientific research should be problem-driven and that the users’ involvement and participation throughout is a fundamental aspect of the science production process (Kirchhoff et al. 2013; Cash and Buizer 2005).

Based on a substantial review of the conditions underpinning the uptake and use of climate information in organisational contexts, Lemos et al. (2012) argue “(…) that to narrow this [usability of scientific information] gap we need to delve deeper into understanding the processes and mechanisms that move information from what producers of climate information (…) hope is *useful*, to what users of climate information (…) know can be applied [and be *usable*] in their decision-making” (Lemos et al. 2012, p.789, emphasis added). Their work offers a framework to understand the main barriers and enablers that can hinder or facilitate the uptake and use of climate information such as SCF in organisations. These are described according to three categories: fit, interplay, and interaction.

*Fit* considers how well users’ perceptions of climate information fit in with the organisational context or culture. The accuracy and reliability of the information being provided, its credibility and salience, and the relevance and usability of that information in the organisation are all factors that can facilitate the uptake of SCF (Cash et al. 2003; Pagano et al. 2002; Lemos and Morehouse 2005; Feldman and Ingram 2009). *Interplay* regards how well this new information relates to, and interacts with, other forms of knowledge or information already available in the organisation. The organisational setting, practises and routines, flexible decision-making processes, in-house expertise and technical capacity, and information seeking are all aspects that can promote the use of SCF in organisational contexts (Lemos 2008; Dilling and Lemos 2011; Bolson and Broad 2013). *Interaction* describes the type and quality of the relationship and collaboration between the producers and the users of that information (Lemos and Morehouse 2005; Rayner et al. 2005; Bolson and Broad 2013; Eden 2011). In this context, the differences in attitudes, priorities and expectations between the scientific and policy communities need to be recognised and addressed in order to bring these groups together (Choi et al. 2005; Hering et al. 2014). In this context, boundary organisations can help mediate the space between these communities or act as knowledge broker by helping to translate and aid communication between them (McNie 2007; Kirchhoff et al. 2013).

## Methods

This study was based on data collected from interviews with organisations across Europe and different economic sectors. Contrary to other methods (e.g. survey) interviews provide a more in-depth understanding of the issues at hand by allowing the interviewees’ to share their knowledge and experiences (May 2011). In addition, alternative participatory methods such as workshops also proved difficult to implement given the geographical scope of the project.

A total of 75 semi-structured interviews were conducted between June 2013 and June 2014. The interview protocol (see Appendix 1) covered questions on the general characteristics of the organisations, the processes of decision-making, the use of weather and climate information including SCF, and how organisations deal with and manage uncertainty in climate information.

This research was part of the EUPORIAS^2^ project whose aim is to demonstrate how SCF can be made usable to decision-makers across a range of European sectors (see Hewitt et al. 2013). The project has a consortium of 60 stakeholders which are organisations in Europe who agreed to be involved in the project from the outset.

Approximately half of the organisations interviewed (*n* = 37) were part of the project’s consortium of stakeholders whilst the rest (*n* = 38) were organisations identified^3^ and approached specifically for this study. In some organisations more than one person was interviewed (or present at the time of the interview) in order to provide information regarding different areas of activities within the organisation (e.g. use of weather and climate information). The majority of the interviewees had leading roles within their organisations (e.g. head or manager of a department) (*n* = 31) or were technical experts in particular areas within their organisation (*n* = 29).

All interviews were audio recorded and transcribed *verbatim* to ensure the quality of the information collected was preserved. We then used qualitative data analysis software (NVivo 10) to code the information and perform thematic analyses of the main themes covered during the interviews: organisation’s characteristics; decision-making and planning activities; use of weather and climate information; use of SCF; and managing uncertainty.

The organisations interviewed were based across different European countries and economic sectors (Table 1). Although a geographical and sectoral representation was aimed at, it proved difficult to engage with and interview organisations in certain European countries (particularly in Eastern Europe) and economic sectors (e.g. insurance, forestry). In some cases this led to an unbalance in terms of geographical representation e.g. tourism interviews were largely conducted in France.

**Table 1:** Number of organisations interviewed per country and sector of activity

	Sector									
Country	Energy	Transport & emergency services	Water	Agriculture	Tourism	Health	Forestry	Insurance	Other	Total
France	6		1		8			2		17
Spain		2	3	4		1		2		12
UK	2	3	2	1		2			1	11
Sweden		2				1	2		2	7
Portugal	1	1	1	2			1			6
Germany	1		1			2	1			5
Italy	1			1		1				3
Denmark		2	1							3
Switzerland				1				1		2
Norway		1	1							2
Belgium					1		1			2
Romania			1							1
Hungary						1				1
Czech Republic	1									1
Cyprus		1								1
Croatia	1									1
Total	13	12	11	9	9	8	5	5	3	75

The organisations interviewed worked across sectors including energy (*n* = 13), transport and emergency services (*n* = 12), water (*n* = 11), agriculture (*n* = 9), tourism (*n* = 9), health (*n* = 8), forestry (*n* = 5), insurance (*n* = 5), and other^4^ (*n* = 3).

The majority of the organisations interviewed were private companies or public organisations (*n* = 25 and *n* = 23, respectively). The remaining organisations were publicly funded organisations (but not part of government), research organisations, international organisations, professional organisations, and consultancies. Some of the organisations interviewed (*n* = 13) acted as intermediary organisations (e.g. research organisations, consultancies) in terms of centralising and/or providing climate information to others (who then act on that information and use it to make decisions). In such instances, the responses provided were mainly based on the interviewees’ role and perceptions of how their clients used SCF. More than half of the organisations interviewed pursued activities at a national level (*n* = 38) and were large organisations with more than 1000 employees (*n* = 31), particularly in the energy sector and transport and emergency services.

## Barriers to the use of seasonal climate forecasts

**Fig. 1:**
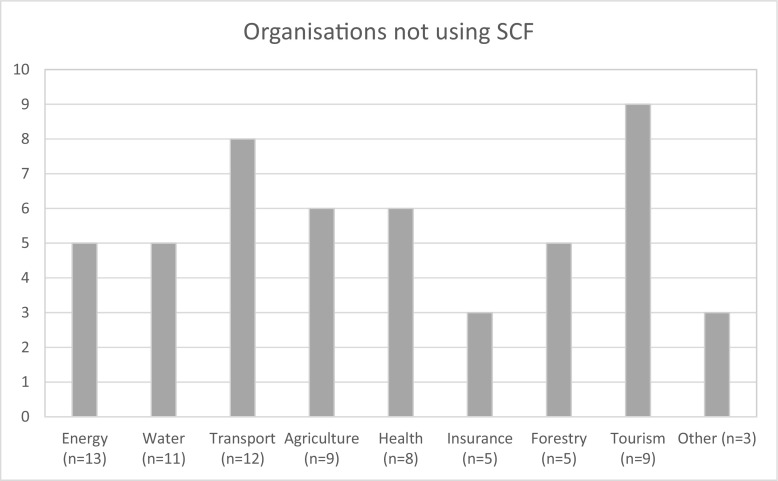
Organisations not using seasonal climate forecasts according to economic sector

The majority of the organisations interviewed did not currently use SCF (*n* = 50; see Fig. 1). These 50 organisations included those working in tourism (*n* = 9), transport and emergency services (*n* = 8), agriculture (*n* = 7), health (*n* = 6), energy (*n* = 5), forestry (*n* = 5), water (*n* = 4), insurance (*n* = 3) and other^1^ (*n* = 3). All of the organisations interviewed in the tourism, forestry, and other sectors did not currently use SCF (Fig. 1).

The main barrier to the use of SCF was the perceived lack of reliability^5^ of these forecasts in Europe (14 of 50 organisations not using SCF). This barrier was often linked to existing perceptions of high levels of uncertainty and lack of accuracy in the forecasts which were overall deemed as not useful in the organisations even as qualitative information i.e. as an indication of potential future climate conditions as expressed in the following quote: “*The few probabilities we get are honestly too uncertain to base some [touristic activities such as] promotion [special offers] or communication. So we don’t use them*.” (IT1^6^).

The lack of relevance of SCF was another major barrier identified (*n* = 10). This was mainly related to situations where the forecast did not fit the organisation in terms of their *modus operandi* i.e. when the organisation was not responsible for pursuing work/activities where the use of SCF could be relevant. The lack of relevance of SCF was also associated with the reactive nature of some of the organisations’ activities to weather and climate conditions (particularly smaller companies in the tourism sector). Many of these organisations did not use climate information on a regular basis and only make use of weather forecasts via online websites. In a few cases, the lack of relevance was also due to the lack of demand from their own clients for this type of climate forecasts.

Another barrier to the use of SCF was the lack of awareness (*n* = 7) of exactly what was available as described in the following quote: *“[We don’t use SCF] because we don’t know what is available, simple as that” (IH1).*

Two of the organisations also mentioned the level of financial investment (and other resources) as well as internal negotiations that would have to be pursued to allow the use of SCF in the organisation.

The tradition of performing historical variability analysis where past observation data is used to perform analysis of future variability was also a barrier in two of the organisations. This tradition was either due to their preference for maintaining existing practices and/or because they perceived this type of analysis to be more reliable for identifying future climate conditions: *“We also use historical information as a substitute for seasonal projections because if we can’t get any seasonal projections that are good enough (…) then the traditional approach we have used is to look at the historical series (…)”* (IW1). For another two organisations lack of understanding of the potential added value of using SCF in their operational models also acted as an obstacle to its use.

In one particular case, the timing of the forecasts (when these were made available to them) also represented a barrier: “*Because we plan a lot of our work about a year and a half out so even if we planned out […] a seasonal forecast that we receive 2 months before isn’t going to be particularly of use*” (ITES1).

The main barriers to the use of SCF in these organisations are listed in Appendix 2. The enablers supporting the use of SCF in the remaining organisations are described below.

## Enablers to the use of seasonal climate forecasts

**Fig. 2:**
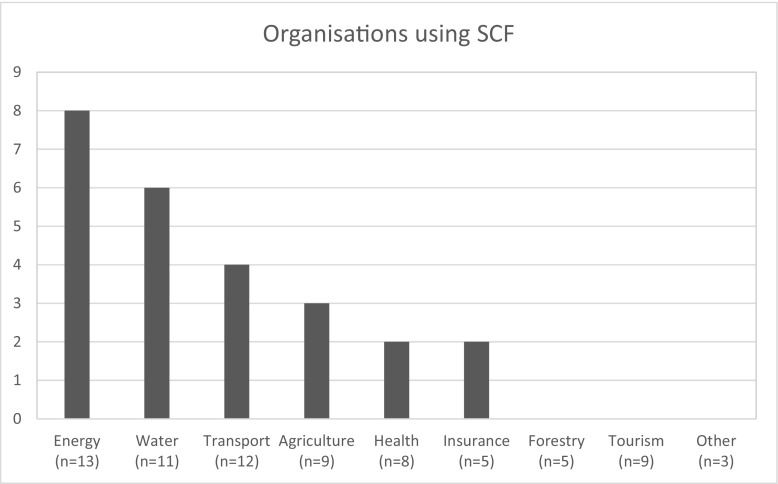
Organisations using seasonal climate forecasts according to economic sector

From the organisations interviewed only 25 used SCF. These included organisations working in the energy (*n* = 8), water (*n* = 6), transport and emergency services (*n* = 4), agriculture (*n* = 3), insurance (*n* = 2), and health (*n* = 2) sectors (Fig. 2).

The main enablers supporting the use of SCF in these organisations were largely related to the relationships with the producers/providers of SCF as well as the level of resources and expertise in the organisation. In many cases, these enablers were present concomitantly in the organisations.

The accessibility to SCF via collaborations and ongoing relationships with the producers was a common factor across the organisations using SCF. However, the type of relationship differed depending on the nature of the organisation and the institutional context in which they are embedded.

One group of organisations was composed of large private companies (9 of the organisations using SCF) that made extensive use of weather information in their operational and planning activities in order to enhance their effectiveness, performance and competitive advantage in the market. These organisations had various collaborations with weather and climate information providers such as the National Meteorological and Hydrological Services (NMHS), the European Centre for Medium Range Forecasts (ECMWF) and other private companies. Those working at the international level also tended to have larger number of collaborations with various climate information providers as described in this quote: “(…) *We get data from suppliers, weather forecast suppliers or agencies. Indeed, as we are present in many countries, we may have many different suppliers (…) that will provide different information. So either raw data, added value data, or forecasts.”* (IE1).

Many of these organisations (particularly private companies) were also equipped with in-house expertise and the necessary resources and capacity to assimilate, process, and use SCF. The perceived advantage of using this type of climate forecasts in a competitive market was also recognised by a few of the organisations. This is well reflected in a quote from an organisation operating in the energy sector: “(…) *most people on this sector (…) look at this kind of information [SCF] whatever the source of the information is and (…) [we] cannot afford not to look at them because others look at it.”* In this same organisation, the interviewee had been recruited more than 10 years ago by that organisation specifically to explore *“(…) if there was any useful information [from] seasonal forecasts for [the company’s] activities (…)” (IE2).*

Two organisations currently use SCF to develop specific products for clients based outside Europe. Higher levels of skill and reliability of SCF, compared to Europe, were another driver for using SCF: “(…) *we use such SCF for two particular clients which are based – or their activity is based – in geographical countries where we can use this kind of information with previsibility [predictability] which is not zero. So we use them for tropical countries (…)*” (IE3).

Another large group (*n* = 9) was primarily composed of government organisations working at the national level and responsible for the provision of public services. In this case, SCF were provided by the NMHS or the ECMWF and were used to help plan their activities and deliver public services in their countries. In addition, many used the SCF which were provided based on existing protocols and public sector collaborations. This is exemplified in the following quote: *“It’s a permanent relationship because the [NMHS] is a governmental organisation and that’s why we (…) use it quite closely (…) and because they are also a governmental organisation. We don’t have normally to pay for this service because it’s a governmental service.”* (IW2).

A smaller group of organisations (*n* = 4) was composed of companies from the public and private sectors mainly working at the national level. In general, these organisations had some contact with the NMHS (normally though a specific contract for weather or climate information provision) but the SCF was normally accessed via the NMHS websites. In this group the main driver for using SCF was largely associated with knowledge-seeking behaviour where SCF was perceived as another potential source of information (even if only used qualitatively). The ways in which these organisations used SCF is described below. The main enablers to the use of SCF identified here are also listed in Appendix 2.

Organisations that used SCF in our sample used them as qualitative information i.e. not formally integrated into any organisational routine.^7^ Instead, the use of forecasts was more akin to a “subjective process” (Bolson and Broad 2013; p. 275) and can be differentiated between moderate and advanced use.

Those using SCF moderately (*n* = 12) use it as information they ‘keep in the back of their minds’ given the [perceived] low reliability of these forecasts. “(…) *we use them, we read them […] we analyse them, but we can’t consider them to have a high level of accuracy and (…) we can’t use it for a professional decision*” (IA1*)*. In such cases, the forecasts tends to be used to provide them with ‘a direction to go’ and to inform a more general opinion on how future conditions may affect the organisation’s operations and activities. Conversely, advanced users (*n* = 13) used SCF to help plan their activities (e.g. maintenance work, emergency planning), managing external contracts, or were in the process of trying to integrate and use SCF operationally. For example, an organisation responsible for roads infrastructure uses SCF to help them manage external contracts: “(…) *We don’t want to be removing asphalt or re-surfacing roads during heavy rainfall, so we have to consider these seasonal variations. We may plan our contracts to come out at a certain time (…) so we can do certain activities under good weather conditions and avoid having to engage the contractors to do re-surfacing in November for example, when we have rain*.” (ITES2). At the time of this study, only one organisation was in the process of integrating SCF into their operational model.

### Remaining barriers to a more efficient use of seasonal climate forecasts

Although SCF is being used (in a qualitative manner and to different extents) there were also limitations that impeded a more effective use of these forecasts in the organisations. The perceived lack of reliability of SCF in Europe was the main barrier to its more effective use (*n* = 5) and although it did not stop these organisations from considering SCF it did prevent them from integrating it into e.g. automated processes such as existing operational models.

The perceived low reliability of SCF allied to issues of capacity and uncertainty also limited the ability to use SCF in some organisations. In such cases, the lack of resources to deal with the low reliability of SCF in terms of having the necessary expertise and/or capacity to perform both pre and post-processing of the data in order to use it operationally limited their ability to use SCF more efficiently.

Another barrier linked to the low reliability of SCF was the uncertainty of forecasts. In three of the organisations interviewed the “(…) *need [for] this type of forecast*” was present given the competitive edge that SCF could provide them in a competitive market (see Section 5 above). However, given the low reliability of the forecasts, these organisations were triangulating SCF data from different sources as a way of reducing the uncertainty in the information provided. It is important to note that these were large organisations with resources and capacity to access various sources of SCF and in-house expertise to compare the forecasts as described in the following quote: “(…) *we compare the forecasts issued by different suppliers […] and then, if this information is contradictory, that is one type of information and, if they are both pointing in the same direction, that is also a type of information*” (IE1).

The timing when the SCF was made available was also considered as a barrier for a couple of the organisations as the information was provided too late to be effectively used in the planning of their seasonal operations and activities.

The content of the information provided was also considered ineffectual by a few organisations (particularly those in the water and health sectors) as they would prefer to have the forecast information translated into potential impacts. In another case, having the SCF provided as 3 months averages did not allow the integration of this information into existing operational models: *“(…) the information today is not adequate for being integrated into [our] models because the timescale and the time step on the information, basically we’re talking about 3 months averages and so on, is really not possible to introduce into our tools (…)”* (IE2).

## On the usability of seasonal climate forecasts in Europe

**Fig. 3:**
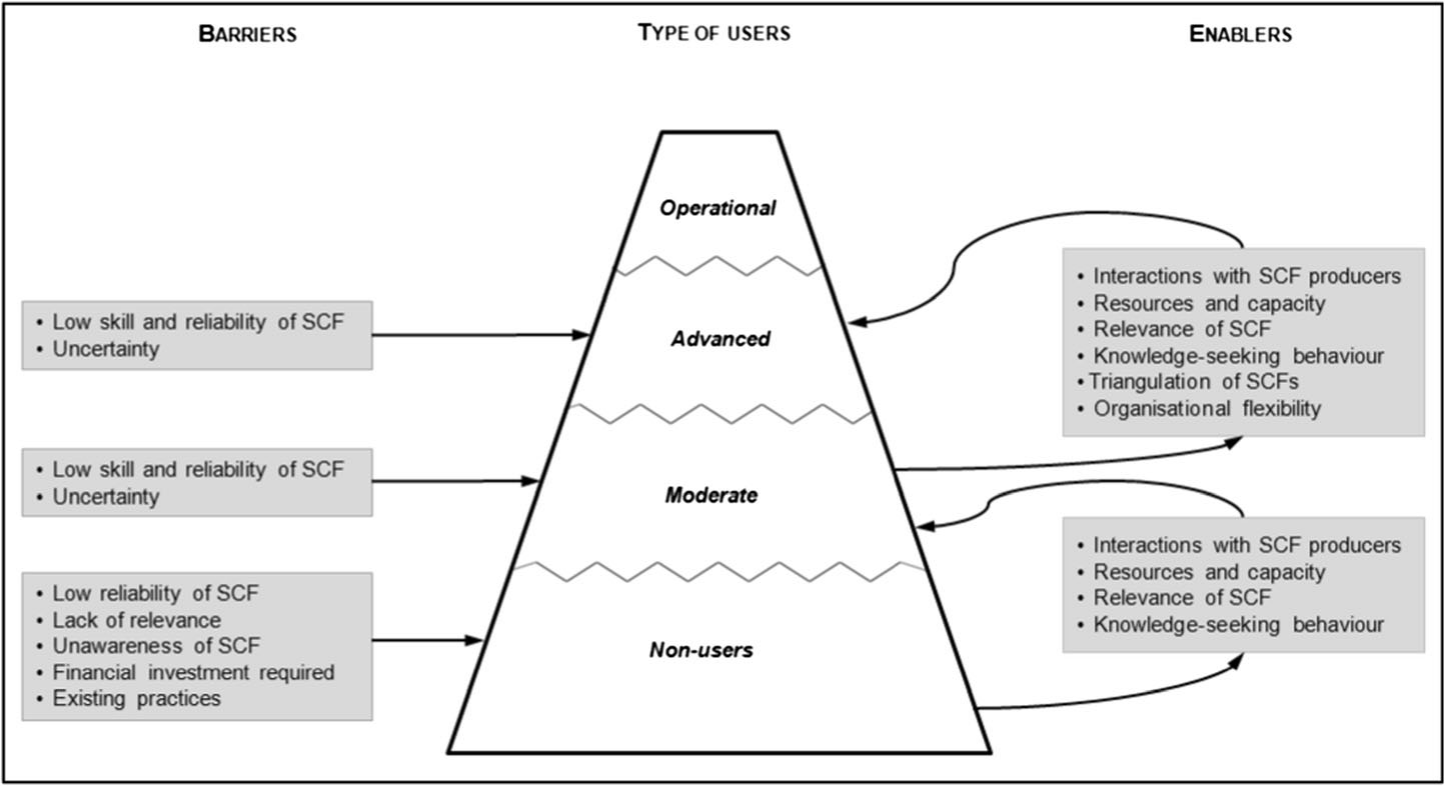
Barriers and enablers to the use of seasonal climate forecasts in Europe

The large majority of the organisations interviewed (*n* = 50) did not use SCF. The main barriers hindering its use related to the quality of the information being provided, the lack of relevance of SCF to the organisation, or due to existing established practices in the organisation (Fig. 3). All of these factors correspond to issues of fit and interplay described by Lemos et al. (2012) (cf. Section 2). The lack of relevance of SCF in the organisations and the level of investment required for the use of SCF were also barriers identified by the non-users of SCF.

Conversely, the use of SCF is still very limited with only one third of the organisations interviewed currently using it (*n* = 25). The main enabler that allowed the use of SCF (to different extents) by these organisations was the *interactions* with the producers (i.e. NMHS, ECMWF, private companies). These interactions were largely based on existing relationships/collaborations where trust and legitimacy had already been built over time between the organisations (Kirchhoff 2013; Dilling and Lemos 2011; Bolson and Broad 2013; Cash et al. 2003). An interesting aspect was that the accessibility to SCF by public sector organisations was mainly pursued through existing protocols between government organisations (e.g. the organisation and NMHS). In such cases, the provision (and use) of SCF aimed at improving public services rather than pursuing private sector goals such as profit maximisation (cf. Steinemann 2006).

Other critical enablers to the use of SCF included the existing level of resources, capacity, and expertise in the organisations (Bolson et al. 2013; Pagano et al. 2002); the relevance of SCF (Lemos et al. 2012); and knowledge-seeking behaviour (Kirchhoff 2013). These enablers were present in both the moderate and advanced users of SCF (Fig. 3).

However, despite that, in all organisations interviewed SCF is still far from being used in an operational way (Fig. 3). In this context, the operational use of SCF is understood as “(…) a specific ordering of work activities across time and place, with a beginning, an end, and clearly identified inputs and outputs: a structure for action.” (Davenport 2013; p.5).

The usability of this type of forecasts in the organisations is still very much compromised by the low skill of SCF in Europe and perceived reliability and uncertainty attached to it. Even those regarded as the more advanced users of SCF were still short of being able to fully integrate SCF into automated processes and operational models (i.e. operational use of SCF). Instead, the maximum level of ‘usability’ achieved by very few organisations was reached through specific enablers (e.g. triangulation of different sources of SCF) that allowed them to adapt and negotiate the use and assimilation of SCF in the organisation. However, such enablers required a level of resources, capacity, and expertise to manage such process as well as an organisational interest in investing in SCF to help optimise their activities. Ultimately, it was the organisational characteristics, resources and conditions of such (larger) organisations that allowed them to (partially) overcome the uncertainty and low reliability of SCF and make use of such information (Lemos and Rood 2010; Lemos et al. 2012; Bolson and Broad 2013).

This study is bound by methodological aspects that influenced the analysis performed and the findings of this research. For example, the interviews conducted were a function of available contacts (both from the EUPORIAS stakeholders and other organisations that were involved through the snowball effect) which ultimately led to a more significant representation by some countries and sectors in this study. In addition, the analysis represents a snapshot in time of the use (or not) of SCF in Europe which is constantly evolving as supply and demand change.

The state of SCF development in Europe is still emerging compared to other regions of the world. As a result, the future of SCF in Europe may be well served by further developing the interface between the science production and the users. Given the low skill of SCF in Europe, it is critical for the users to have a more prominent and active role in the development of this type of forecasts. In addition, and as we have shown, the most common and significant enabler to the use of this type of forecasts in Europe are the interactions with the producers/providers of SCF. As such, developing such interfaces that allow for collaborations between the actors involved in the production, provision and use of SCF can be both critical and have multiple benefits. For example, it would help users understand how and if uncertain and probabilistic information such as SCF can be best adapted to their needs (e.g. how leading organisations are doing it) and allow them to feedback their needs in the development of scientific information and thus push for ‘problem-driven’ science to be developed. On the production and provision side it would also allow them to take stock and tailor (existing and new) products according to users’ needs and requirements which would potentially lead to an increase uptake and use of SCF.

This interface space points towards a need for dedicated boundary organisations or knowledge broker organisations capable of opening up the usability of this data, making the information, resources and techniques currently used by only a few large organisations more widely available to others who may also benefit from using SCF (cf. McNie 2007; Reinecke 2015). In Europe, the need for such specialised organisations in the context of SCF has been recognised (see Bruno Soares and Dessai 2015) although such initiatives to date have been mostly pursued in the context of adaptation to long-term climate change (Reinecke 2015). Such advances would also contribute significantly to the emerging context of climate services development in Europe and the potential role that SCF can play in it.

## Conclusions

The use of SCF in Europe is relatively new compared to other regions where the uptake of this type of forecasts has a longer history. In order to understand the current usability of SCF we interviewed 75 organisations working across a range of economic sectors in Europe. This allowed us to determine the existing barriers to the use of SCF as well as the main drivers underpinning the use of SCF in the organizations.

Our findings have shown that the main barriers to the use of SCF in organisations in Europe were largely associated to the low reliability and skill of SCF in Europe as well as with other non-scientific factors such as the lack of relevance of SCF in the organisation, the lack of awareness of what is available, and the level of investment and resources required to use these forecasts. This demonstrates that the limited use of SCF amongst organisations in Europe is also related to other institutional factors that go beyond the low reliability of SCF. As such, future efforts to increase the usability of this type of forecasts in Europe should also focus on those non-technical aspects that may also represent significant barriers to its use (e.g. unawareness of SCF, the level of financial resources required to use SCF).

The main enablers supporting the use of SCF were largely linked to long-term interactions and relationships with the producers of SCF although these tend to be of a different nature depending on the type of organisation (private/public sector). Access to organisational resources, capacity and expertise were also critical factors for the use of SCF. In some cases, high levels of resources and expertise allowed organisations to work with different SCF and manipulate them to apply it in their decision-making.

These findings also confirm what has been experienced in other regions and countries regarding barriers and enablers to the use of SCF (see e.g. Lemos et al. 2012; Dilling and Lemos 2011; cf. Section 2). Nonetheless, this study represents the first empirical assessment of this type in Europe and, as a result, it should be considered when thinking of how science that works for users in Europe can be developed. For example, by fostering new interfaces and ways of interacting with the SCF producers and/or with intermediary organisations (i.e. boundary organisations or knowledge brokers) in order to support the uptake of SCF in Europe.

The outcomes of this study should be considered not only in the context of how to increase and improve the usability of SCF but also in the wider context of climate services development in Europe. Recent initiatives and efforts to advance a climate services market in Europe (see European Commission 2015) raises important questions regarding the development of the climate science such as SCF but more fundamentally how that data and information will fit into, and enhance, the decision-making processes of end-users in Europe.

Although at an early stage, this paper captures the issues at this point in time and highlights the importance of developing more usable science, by developing the interface that can support organisations explore the value of uncertain science in helping them to cope with climate variability.

## Electronic supplementary material

Below is the link to the electronic supplementary material.

ESM 1 (DOCX 17 kb)

ESM 2 (DOCX 71 kb)

## Footnotes

1 For more information on these Global Producing Centers see: http://www.wmo.int/pages/themes/climate/global_producing_centres.php

2 EUPORIAS is an EU FP7 project and stands for European Provision of Regional Impacts Assessments on Seasonal and Decadal Timescales project. For more on EUPORIAS see: www.euporias.eu

3 In some cases, the interviewees suggested other contacts/organisations that could be of interest to the remit of this research project (snowball effect).

4 These interviews were conducted with public sector organisations working on environmental issues.

5 The term *reliability* is used here as a synonym of trustworthiness and, as a result, it can be mapped onto a number of other technical concepts such as *skill*, *reliability*, and *sharpness*. In this paper, we mainly refer to the perceived reliability of SCF by the interviewees unless otherwise stated.

6 IT = Interview in the tourism sector; IH = Interview in the health sector; ITES = Interview in transport and emergency services; IE = Interview in the energy sector; IW = Interview in the water sector; IA = Interview in the agriculture sector. The numbers correspond to an internal code used to identify each interview.

7 By routines we mean the integration of information into operational models and automated decision processes.
