# Supplementary material for: Barriers and enablers to the use of seasonal climate forecasts amongst organisations in Europe
Source: Clim Change. 2016 Apr 15;137(1):89–103. doi: 10.1007/s10584-016-1671-8 (PMC7154867; doi:10.1007/s10584-016-1671-8)
Supplement: Supplementary file 1 — (DOCX 17 kb) [file 10584_2016_1671_MOESM1_ESM.docx]

## Appendix 1 – Interview protocol

1. General information

1.1. Can you tell me a bit about your organisation and the work it does? (Including sector and type of activities it performs).

1.2. How would you classify your organisation? (e.g. government, international organisation, private company, consultancy, research institution, non-governmental organisation, other).

1.3. At what geographical scale does your organisation operate? (e.g. international, European, national, regional, local level).

1.4. How many employees does your organisation have?

- Up to 10 employees;
- Up to 50 employees;
- Up to 250 employees;
- More than 250 employees.

1.5. What is your role in the organisation?

1.6. How is your organisation and sector governed? e.g., by government, independent regulators, industry standards, EU directives, consumers, other.

2. Decision-making processes in the organisation

*This section includes questions regarding decision-making in the organisation namely timescales for decisions, the type of information used to make decisions, and how the organisation plans for the future.*

2.1. How does your organisation plan for the future? What are the main activities that need to be planned beyond a month?

2.2. Are there activities that need to be planned at longer timescales? For example, 3 months, 6 months, 1 year, 5 years, 10 years, more than 10 years?

2.3. What are the critical factors that need to be considered or accounted for when you plan for those activities? (e.g., consumer demand, weather, commodity price, regulatory approval, other)

2.4. When planning for the future how do you account for uncertainty? Do you use any tools or information that help you account for that uncertainty in your decision-making such as scenario analysis, probabilistic risk assessment, etc.?

2.5. What are the main challenges in accounting for that uncertainty in your decision-making processes?

2.6. What type of information does your organisation use to make decisions (e.g. social data, economic data, etc.)? What are the main channels through which that information is obtained e.g. reports, TV, colleagues, smartphone applications, radio?

3. Use of weather and climate information

*This section covers questions on the organisation’s sensitivity to weather and climate and the use and provision of climate information in the organisation. Interviewer: you can use the diagram provided at the end of this protocol to briefly explain the differences between weather and climate change and related types of forecasts if needed.*

3.1. Is your organisation sensitive to weather (e.g., high/low rainfall, temperature, wind, snow) and its impacts (e.g., droughts, floods)? Please describe how your organisation’s activities are affected (positively and negatively) by such events.

3.2. Does your organisation use weather/climate information to make decisions?

- If yes, please describe the information used (e.g. weather forecasts; past observations; seasonal climate forecasts, climate change projections, climate impacts) and the type of activities and decisions being planned;
- If no, please describe why your organisation doesn’t use weather/climate information (go to question 3.8 below).

3.3. Climate information can also be provided in the form of indices describing the potential impacts of climate. Examples of this type of indices include:

- Heating Degree Days which corresponds to a sum of cold temperature days and therefore indicates the effort required to heat buildings;
- Growing Degree Days which corresponds to temperature sum above a given threshold and can be used as an index for plant productivity;
- Heavy precipitation indices which give an indication for possible flooding;
- Storm indices which summarize information on wind strength and give an estimation of possible damages.

Does your organization use this type of indices?

- If yes, how is this information used to make decisions?
- If not, could you think of any helpful measures or indices that could be useful to your organization?

3.4. How important is weather/climate information compared to other types of information that influence decisions in your organisation?

3.5. Where does your organisation obtain its weather/climate information (including information on climate impacts)? Does your organisation pay for this information?

3.6. What sort of relationship does your organisation have with the weather/climate information provider(s) of that information e.g., client relationship, collaborative relationship, etc.?

3.7. Is this weather/climate information processed/tailored before being used?

- If yes, please describe how and by whom (e.g. climate service provider, consultancy, someone in your organisation?)
- If no, would it be helpful to have particular climate information tailored? What kind of climate information?

3.8. Does your organisation provide climate information to others?

- If yes, please describe the type of information provided, the user, and the purpose of such provision;
- Is this a new or long-established activity?

3.9. Is there climate information that is currently not available and that would be useful to have in your organisation or sector? Please describe it and how it would help your organisation or sector.

3.10. In your opinion, which weather/climate products should be provided as a public service (and therefore freely available) and which should be a private service (i.e. with a cost attached)?

4. Use of seasonal to decadal (S2D) climate information

*This section includes questions on the use of S2D climate information in the organisation and their expectations of what this information can provide.*

4.1. Are you aware of seasonal climate information? If so, can you describe what seasonal climate information is in your own words?

*Interviewer: you can use the example of a seasonal forecast available on the Content Management System if you need to explain it to the interviewee.*

4.2. Does your organisation use seasonal climate information such as seasonal or monthly forecasts?

- If yes, please describe the type of information used with regard to:

o Activities and decision-making processes it informs;

o Who provides that information;

oThe reasons why that information is used in your organisation e.g. availability, usefulness.

- If no, please describe the reasons for not using this type of information (e.g. lack of predictability, inadequacy of information provided, costs for accessing such data, not aware).

o If this information was available to you, how would your organisation use this information?

o Which type of seasonal/monthly information would be useful to your organisation? (e.g. 3-month temperature forecast)

o Would your organisation be willing to pay for this information?

4.3. Does your organisation use annual/decadal climate information?

- If yes, please describe the type of information used with regard to:

o Activities and decision-making processes it informs;

o Who provides that information;

o The reasons why that information is used in your organisation e.g. availability, usefulness.

- If no, please describe the reasons for not using this type of information (e.g. lack of predictability, inadequacy of information provided, costs for accessing such data, not aware).

o If this information becomes more widely available, what conditions would have to be in place for your organisation to start using this climate information in its decision-making?

o If so, which type of information would be useful to your organisation? If known, please describe the required climate variable(s) and the spatial/time resolution.

o And why would you use this information? (e.g., credibility, improve decision-making, cost);

o Would your organisation be willing to pay for this information if it becomes more widely and readily available?

4.4. Who do you think should be responsible for producing and disseminating seasonal and decadal climate information?

4.5. Based on your past experience or your perception how reliable are these predictions?

4.6. If seasonal and decadal forecasts become more widely available in the future, which do you think should be provided as a public service (i.e. available free of charge) and which should be a private service (i.e. with a cost attached)?

4.7. Are you aware of any other organisations that are using or should be using S2D climate information? If so, can you describe how and why they are using this information. Can you please provide me with their contact details?

5. Dealing with uncertainty

*This section covers issues regarding the uncertainty of climate information and how the organisation deals with it.*

5.1. S2D forecasts usually come with information about the degree of uncertainty in the forecast.

- If not a current user of S2D forecasts: How useful would this information be to you? How would you use it in your decision-making?
- If a current user of S2D forecasts: How do you deal with uncertainty in S2D forecasts? Do you only use them given a certain signal strength (or confidence level...)? When using such forecasts, do you check their skill assessment?

5.2. There are different ways of representing the uncertainty in forecasts such as using verbal descriptions, numerical estimates and/or graphics. How would you like to receive information about forecast uncertainty? And why would you prefer this method of representation?
