# Supplementary material for: Barriers and enablers to the use of seasonal climate forecasts amongst organisations in Europe
Source: Clim Change. 2016 Apr 15;137(1):89–103. doi: 10.1007/s10584-016-1671-8 (PMC7154867; doi:10.1007/s10584-016-1671-8)
Supplement: Supplementary file 2 — (DOCX 71 kb) [file 10584_2016_1671_MOESM2_ESM.docx]

| Appendix 2 – Main barriers and enablers to the use of seasonal climate forecasts in the organisations. | |
| --- | --- |
| **Main barriers** | **Main enablers** |
| - Perceived lack of reliability of SCF - Lack of relevance of SCF for the organisation’s activities - Lack of awareness of SCF - Level of financial investment and resources required to use SCF in the organisation - Tradition of performing historical variability analysis (due to preference to maintain existing practise or perception of reliability of this type of analysis) - Lack of understanding regarding the added value of SCF - Timing of the forecasts | - Ongoing relationships with the producers/providers of SCF and accessibility to these forecasts (e.g. via protocols between governmental organisations) - Level of resources and expertise in the organisation - Perceived advantage of using SCF particularly in the private sector - Ability to develop specific products in regions where SCF reliability is higher (e.g. tropics) - Knowledge-seeking behaviour |
